# Disheveled3 enhanced EMT and cancer stem-like cells properties via Wnt/β-catenin/c-Myc/SOX2 pathway in colorectal cancer

**DOI:** 10.1186/s12967-023-04120-8

**Published:** 2023-05-05

**Authors:** Zhengguang Li, Zhirong Yang, Wei Liu, Wanglong Zhu, Lan Yin, Zhenyu Han, Yu Xian, Jie Wen, Hualong Tang, Xinyue Lin, Yuhan Yang, Jingyi Wang, Kun Zhang

**Affiliations:** 1 Oncology Department of Chengdu Seventh People’s Hospital, Chengdu, China; 2 Pathology Department of Deyang People’s Hospital, Deyang, 618000 China; 3 The Second Affiliated Hospital of Chengdu Medical College, China National Nuclear Corporation 416 Hospital, Chengdu, 61051 China; 4 School of Biological Sciences and Technology, Chengdu Medical College, Chengdu, 610500 China

**Keywords:** Disheveled, Stem cell, Epithelial-to-mesenchymal transition, Colorectal cancer, SOX2

## Abstract

**Background:**

Epithelial-to-mesenchymal transition (EMT) and cancer stem-like cells (CSLCs) play crucial role in tumor metastasis and drug-resistance. Disheveled3 (DVL3) is involved in malignant behaviors of cancer. However, the role and potential mechanism of DVL3 remain elusive in EMT and CSLCs of colorectal cancer (CRC).

**Methods:**

UALCAN and PrognoScan databases were employed to evaluate DVL3 expression in CRC tissues and its correlation with CRC prognosis, respectively. Transwell, sphere formation and CCK8 assay were used to assess metastasis, stemness and drug sensitivity of CRC cells, respectively. Western blotting and dual luciferase assay were performed to analyze the protein expression and Wnt/β-catenin activation, respectively. Lentiviral transfection was used to construct the stable cell lines. Animal studies were performed to analyze the effect of silencing DVL3 on tumorigenicity and metastasis of CRC cells in vivo.

**Results:**

DVL3 was overexpressed in CRC tissues and several CRC cell lines. DVL3 expression was also higher in CRC tissues with lymph node metastasis than tumor tissues without metastasis, and correlated with poor prognosis of CRC patients. DVL3 positively regulated the abilities of migration, invasion and EMT-like molecular changes in CRC cells. Moreover, DVL3 promoted CSLCs properties and multidrug resistance. We further identified that Wnt/β-catenin was crucial for DVL3-mediated EMT, stemness and SOX2 expression, while silencing SOX2 inhibited DVL3-mediated EMT and stemness. Furthermore, c-Myc, a direct target gene of Wnt/β-catenin, was required for SOX2 expression and strengthened EMT and stemness via SOX2 in CRC cells. Finally, knockdown of DVL3 suppressed tumorigenicity and lung metastasis of CRC cells in nude mice.

**Conclusion:**

DVL3 promoted EMT and CSLCs properties of CRC via Wnt/β-catenin/c-Myc/SOX2 axis, providing a new strategy for successful CRC treatment.

## Introduction

Colorectal cancer (CRC) is reported as the second most prevalent malignancy in China, and remains the second leading cause of cancer-related mortality worldwide [1, 2]. Distant metastasis and drug-resistance are main cause of CRC-associated mortalities [3]. Although great improvements in surgical operation and combined systemic drug therapy have prolonged survival to some extent, the prognosis of CRC patients remains poor [4, 5]. Thus, it is urgent to identify the underlying mechanisms of metastasis and drug-resistance for improving the treatment of CRC.

Epithelial-to-mesenchymal transition (EMT) contributes to various cancer progression, rendering tumor cells invasive and able to metastasize distant organs [6]. EMT is a cellular process in which cells lose their epithelial characteristics and acquire mesenchymal features. Epithelial cancer cells undergoing EMT discard tight junctions, cell polarity and cytoskeletal reorganization, and become more migratory and invasive [7, 8]. Increasing evidence has revealed that cancer stem-like cells (CSLCs) are also involved in malignant progression of cancer. CSLCs are a very small population of cells with stemness in situ tumor tissue [9]. Due to their acquisition of normal stem cell properties including self-renewal, multipotency, dormant state, resistance to apoptosis, and high metastatic potential, CSLCs are currently considered as the chief culprit of tumor occurrence, metastasis and drug-resistance [10, 11]. Moreover, a growing body of evidence suggests that EMT is crucial for the enrichment of CSLCs, which is believed to be responsible for poor prognosis of cancer patient [12, 13]. Therefore, the identification of specific molecules targeting EMT and CSLCs may be an effective way to improve CRC treatment .

EMT and CSLCs are regulated by altered signaling pathways including Wnt and TGF-β signaling [14–16]. Disheveled (DVL) is a critical hub of receptors and downstream components of Wnt signaling [17]. Notably, even without Wnt ligand, DVL was effective in activating Wnt/β-catenin signaling [18]. Not only that, but DVL bridges the cross-talking between Wnt and TGF-β pathways [19]. Recently, DVL is also found to be overexpressed in malignant and recurrent tumor [20, 21]. Nevertheless, the role and mechanism of DVL in EMT and CSLCs of CRC remain to be elucidated. Here, we found that DVL3 was upregulated in CRC tissue and be closely related to poor prognosis of CRC patient. In addition, knockdown of DVL3 inhibited CSLCs properties and EMT that are crucial for metastasis and chemoresistance in CRC cells. Mechanistically, this study clarified that DVL3 promoted EMT and stemness of CRC via Wnt/β-catenin/c-Myc/SOX2 pathway.

## Materials and methods

### Database (DB) analysis

The expression of DVL3 in CRC were analyzed by using data obtained from the UALCAN database (http://ualcan.path.uab.edu/analysis.html), which has enabled users to evaluate protein-coding gene expression across 33 types of cancers [22]. We also employed PrognoScan database (http://dna00.bio.kyutech.ac.jp/PrognoScan/index.html) to assess the relationship between DVL3 expression and CRC prognosis including overall survival (OS), disease free survival (DFS) and disease-specific survival (DSS) [23].

### Cell culture

HCT-8 and SW620 cells (Shanghai Bogoo Biotechnology, Shanghai, China) were cultured in RPMI-1640 with 10% fetal bovine serum (Gibco, Carlsbad, CA, USA), 100 U/mL penicillin and 100 µg/mL streptomycin, and maintained at 37 °C with 5% CO_2_. When cells reached 80‒90% confluency, then were detached with 0.25% trypsin to passage. All cells were verified to be pollution free of mycoplasma, and identified by STR profiling before the experiments.

### Migration and invasion assays

Cell migration was examined by using transwell assay. Briefly, CRC cells (2.5 × 10^4^ cells) were resuspended in 200 µL FBS-free RPMI-1640 medium, and seeded in the top chamber of each transwell (Corning, New York, NY, USA). The medium supplemented with 10% FBS was added in the lower chamber. The cells were incubated at 37 ℃ in a humidified 5% CO_2_ atmosphere for 24 h, and then fixed and stained. The number of cells in six randomly selected fields was counted under microscope. Cell invasion assay was conducted similarly, except that the upper chamber was coated with Matrigel (BD Biosciences, San Jose, CA, USA) for 6 h before cells were seeded. After 48 h seeding, the rest protocol was implemented in a similar manner to the migration assay.

### Western blot analysis

The procedures for western blotting analysis and protein visualization were executed as described previously [24]. Primary antibodies against DVL3 and β-catenin were purchased from Santa Cruz Biotechnology (Santa Cruz, CA, USA). Primary antibodies against E-cadherin, ZO-1, N-cadherin, Vimentin, Snail, CD44, CD133, SOX2, c-Myc were gained from Cell Signaling Technology (Danvers, MA, USA). Anti-β-actin (Santa Cruz, CA, USA) was used as a loading control. The horseradish peroxidase (HRP)-conjugated secondary antibody was obtained from ZSGB-bio (Peking, China). The signals were checked by an enhanced chemiluminescence detection kit(Invitrogen, Carlsbad, CA, USA) and Bio-Rad Molecular Imager (Hercules, CA, USA). The relative expression of above proteins was normalized against β-actin using Image J analysis.

### Lentiviral vector, plasmids, shRNA, and transfection

The cDNAs of DVL3, c-Myc and SOX2 were subcloned into pcDNA3.1 to generate plasmid. Lipofectamine 3000 transfection reagent (Invitrogen, Long Island, USA) was employed to transiently transfect the plasmid or shRNA for upregulating or reducing expressions of DVL3, c-Myc and SOX2. To generate stable knockdown and overexpression cells lines, the cells were transfected with lentivirus-DVL3 (LV-DVL3), LV-shDVL3, LV-shSOX2, LV-c-Myc or the control (LV-con). And then, we selected cells that transduced with puromycin (GeneChem, Shanghai, China).

### Tumor sphere formation assay

CRC cells were harvested and counted. Then 1 × 10^4^ cells were seeded in 6-well ultra-low attachment plates per well (Corning, New York, NY, USA) in serum-free DMEM-F12 medium (Gibco, Carlsbad, CA, USA) supplemented with EGF (20 ng/mL, Beyotime, Shanghai, China), b-FGF (10 ng/mL, Beyotime, Shanghai, China) and B27 (1:50 dilution, Beyotime, Shanghai, China). Subsequently, cells were cultured at 37 °C in an atmosphere containing 5% CO_2_ to form tumorsphere. After 7–10 days, the images of spheres were captured with an inverted microscopy at a magnification of ×100 or ×200. The number of spheres with diameters of > 50 μm was counted and plotted using ImageJ software.

### Cell viability assay

Cell Counting Kit-8 (CCK-8, Hanbio, Shanghai, China) was used to evaluate cell viability according to the instructions. Briefly, cells (5 × 10^3^/well) were seeded in a 96-well plate. After 24, cells were treated with the indicated concentrations of vincristine and oxaliplatin (Sigma-Aldrich, St Louis, USA) for 48 h of incubation. At the end of experiments, CCK-8 reagent was placed into medium at 100 µL/well and incubated for an additional 1 h at 37 °C. Absorbance of each well was examined at 450 nm using a microplate reader (Bio-Rad, Hercules, CA, USA). Vincristine and oxaliplatin concentrations that achieved 50% growth inhibition (IC50) were calculated from cell survival curves using the Bliss method.

### Dual luciferase assay

The transcriptional activity of Wnt/β-catenin signaling was assessed using TOPflash and FOPflash reporters (Upstate Biotechnology, Lake Placid, NY, USA). TOPflash includes six wildtype β-catenin/TCF-binding sites upstream of a luciferase reporter, while FOPflash containing six mutant β-catenin/TCF-binding sites is employed as the control of TOPflash [25]. When cells reached 70–90% confluency in 24-well plates, pcDNA3.1-DVL3 or shDVL3 was co-transfected with TOPflash plus pRL-SV40 or FOPflash plus pRL-SV40 into CRC cells for 48 h. Subsequently, we used to a dual-luciferase assay (Promega Corporation, Madison, WI, USA) to test the activities of TOPflash and FOPflash reporters. The reporter activity of each sample was normalized against Renilla reporter pRL-SV40 (Promega Corporation, Madison, WI, USA) activity to monitor transfection efficiency.

### Tumor xenograft and metastasis in experimental animals

All animal experiments were approved by the Institutional Animal Care and Use Committee of Chengdu Medical College. 5-week-old BALB/c nude mice were purchased from Beijing Charles River Laboratories. For subcutaneous transplanted model, HCT-8 cells stably transfected with LV-shNC and LV-shDVL3 were diluted in 100 µL RIPM-1640, and injected subcutaneously in the rear flank fat pad of the nude mice (2 × 10^6^ cells per mice). When the tumor was visible, tumor growth was measured every three days using calipers, with the tumor volume (mm^3^) calculated using the following formula: V = L (length) × W (width)^2^/2. All the mice were sacrificed before they reached the ethical endpoint. And then, the subcutaneous tumors of mice were harvested and weighed. Followingly, tumors tissues were used to extract total protein for western blotting analysis. For tumor metastasis model, the mice were intravenously injected with 2 × 10^6^ HCT-8 cells stably transfected with LV-shNC and LV-shDVL3 via lateral tail vein. After 4 weeks, the mice were euthanized, and lung tissues were immediately collected and photographed, followed by counting of the pulmonary metastatic nodules in each lobe. Lung metastasis tumor was also identified by hematoxylin and eosin (H&E) staining.

### Statistical analysis

All experiments were independently repeated three times or specified at figure legends. Data were showed as mean ± standard deviation (SD). The conformity to the normal distribution was checked by Shapiro-Wilk test. Based on the data obeying normal distribution, the student’s t-test (two-tailed) or the analysis of one-way ANOVAs was employed to examined the differences. All statistical analysis was performed by GraphPad Prism 6.0 (GraphPad Software Inc., San Diego, CA, USA). A value of *P* ≤ 0.05 was recognized as significant.

## Results

### DVL3 overexpression was positively associated with poor prognosis of CRC

**Fig. 1:**
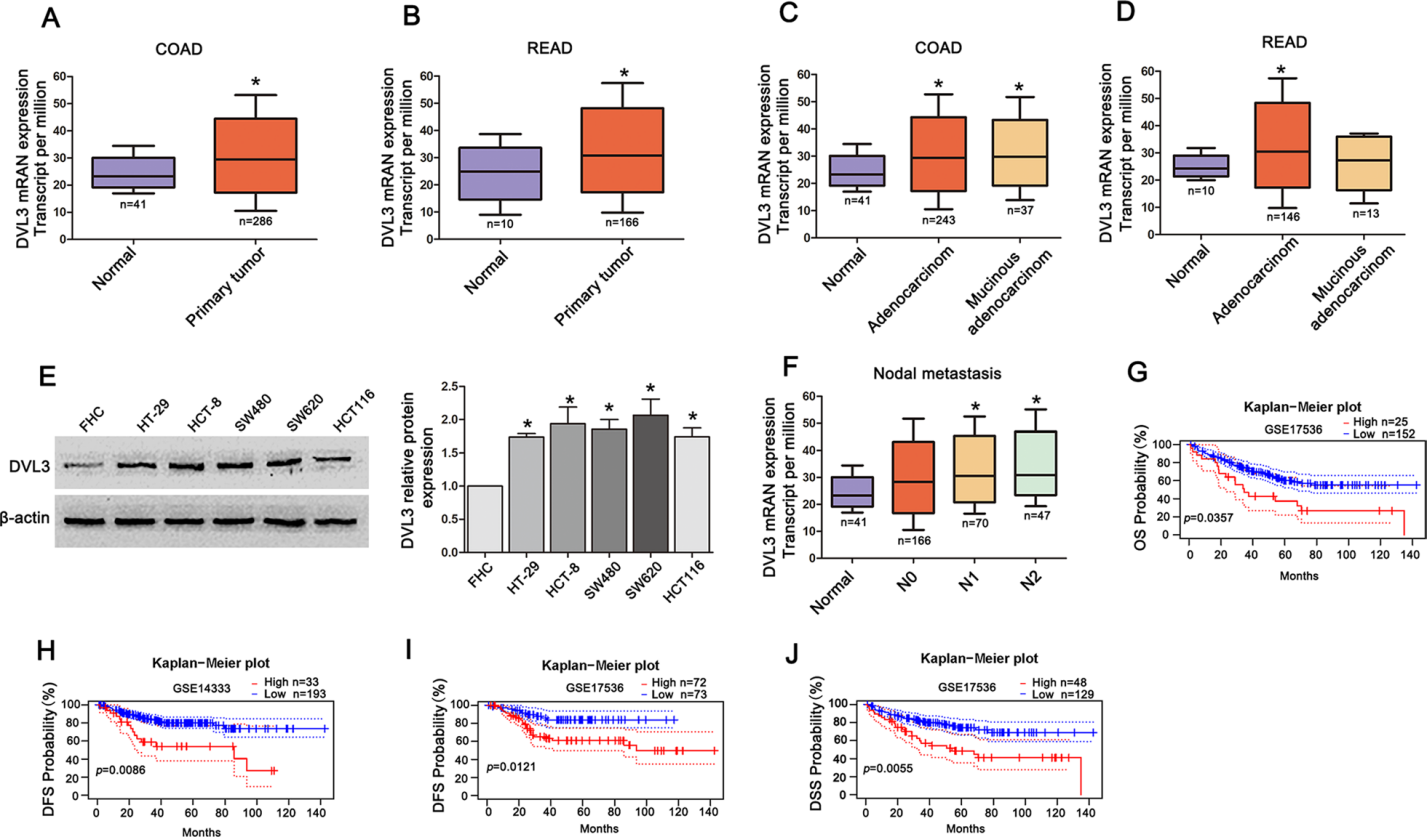
DVL3 overexpression was positively associated with poor prognosis in CRC patients.** A**–**D** Data from the UALCAN database showed that DVL3 was elevated in CRC tissues compared to normal controls. **E** The result of western blotting revealed that DVL3 expression was stronger in HT-29, HCT-8, SW480, SW620 and HCT116 cells than that in FHC cells. **F** DVL3 was upregulated in N1 and N2 stage of CRC nodal metastasis compared to N0 stage in data from the UALCAN database. **G** CRC patients with high DVL3 expression presented shorter overall survival (OS), **H**,** I** disease free Survival (DFS), **J** disease-specific survival (DSS) than patients with Low DVL3 expression, based on data from PrognoScan database. *P ≤ 0.05

In view of the relationship between DVL3 and CRC progression, we examined the expression of DVL3 in UALCAN database (http://ualcan.path.uab.edu/analysis.html). The results indicated that the mRNA level of DVL3 was increased in CRC tissues including adenocarcinoma and mucinous adenocarcinoma, compared to normal tissues (Fig. 1A–D). The western blotting examination showed that the protein level of DVL3 was more abundant in CRC cell lines (HT-29, HCT-8, SW480, SW620 and HCT116) than normal colorectal epithelial cells FHC (Fig. 1E). Moreover, the higher mRNA level of DVL3 was found in N1 and N2 stage of CRC nodal metastasis in contrast with N0 stage (Fig. 1F). Furthermore, Kaplan-Meier analysis from PrognoScan database (http://dna00.bio.kyutech.ac.jp/PrognoScan/index.html) showed that elevated expression of DVL3 was negatively related to overall survival (OS), disease free survival (DFS) and disease-specific survival (DSS) of CRC (Fig. 1G–J). Above results suggested that CRC patients with overexpression of DVL3 suffered from poor prognosis.

### DVL3 heightened metastatic potential of CRC cells

**Fig. 2:**
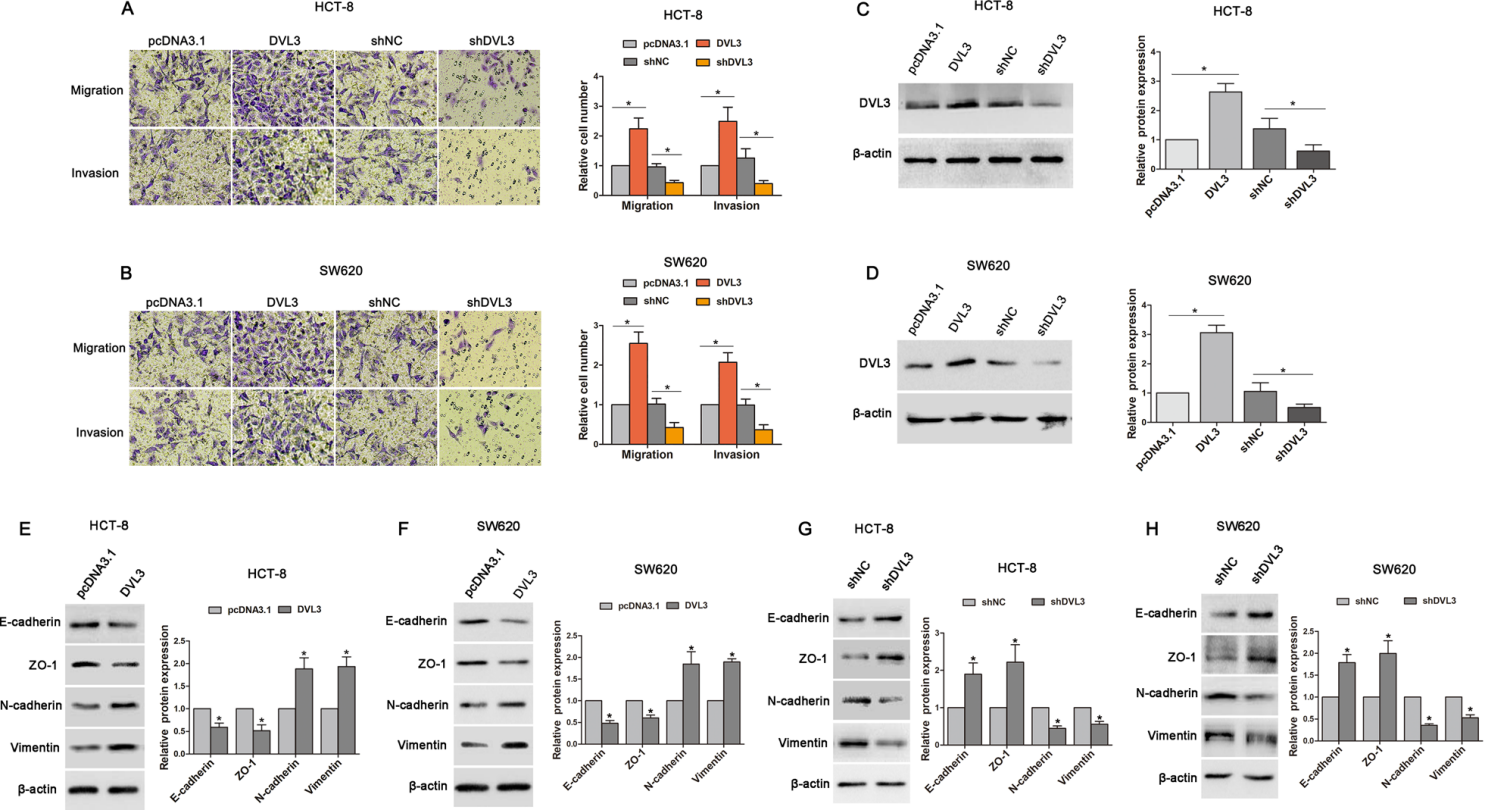
DVL3 enhanced metastatic potential of CRC cells. Transwell assays were used to determine the potential migration and invasion of HCT-8 (**A**) and SW620 (**B**) cells transfected with pcDNA3.1, pcDNA3.1-DVL3, shNC or shDVL3 for 72 h. **C**, **D** The transfection efficiency were confirmed by western blotting. The expressions of E-cadherin and ZO-1, N-cadherin and Vimentin were detected by western blotting in HCT-8 (**E**) and SW620 (**F**) cells transfected with pcDNA3.1 or pcDNA3.1-DVL3 for 72 h, as well as in HCT-8 (**G**) and SW620 (**H**) cells transfected with shNC or shDVL3 for 72 h. *P ≤ 0.05

To explore the role of DVL3 in CRC progression, we determined the effect of DVL3 on migration and invasion of HCT-8 and SW620 cells. DVL3 recombinant vector was transfected into CRC cells, the result of transwell assay showed that DVL3 overexpression increased migratory and invasive potential in HCT-8 and SW620 cells. In contrast, silencing DVL3 reduced the migration and invasion of HCT-8 and SW620 cells transfected with shDVL3 (Fig. 2A–D). We further examined the impact of DVL3 on EMT which plays a critical role in metastasis. In HCT-8 and SW620 cells transfected with DVL3 recombinant vector, epithelial markers (E-cadherin and ZO-1) were downregulated, while the mesenchymal markers (N-cadherin and Vimentin) were upregulated (Fig. 2E, F). To validate that the expressions of E-cadherin, ZO-1, N-cadherin and Vimentin were controlled by DVL3, shDVL3 was transfected into HCT-8 and SW620 cells. As predicted, the protein levels of E-cadherin and ZO-1 were increased by shDVL3, while N-cadherin and Vimentin were decreased (Fig. 2G, H). These results suggested that DVL3 positively regulated metastasis and EMT-like molecular changes in CRC cells.

### DVL3 was involved in TGF-β1-induced EMT of CRC cells

**Fig. 3:**
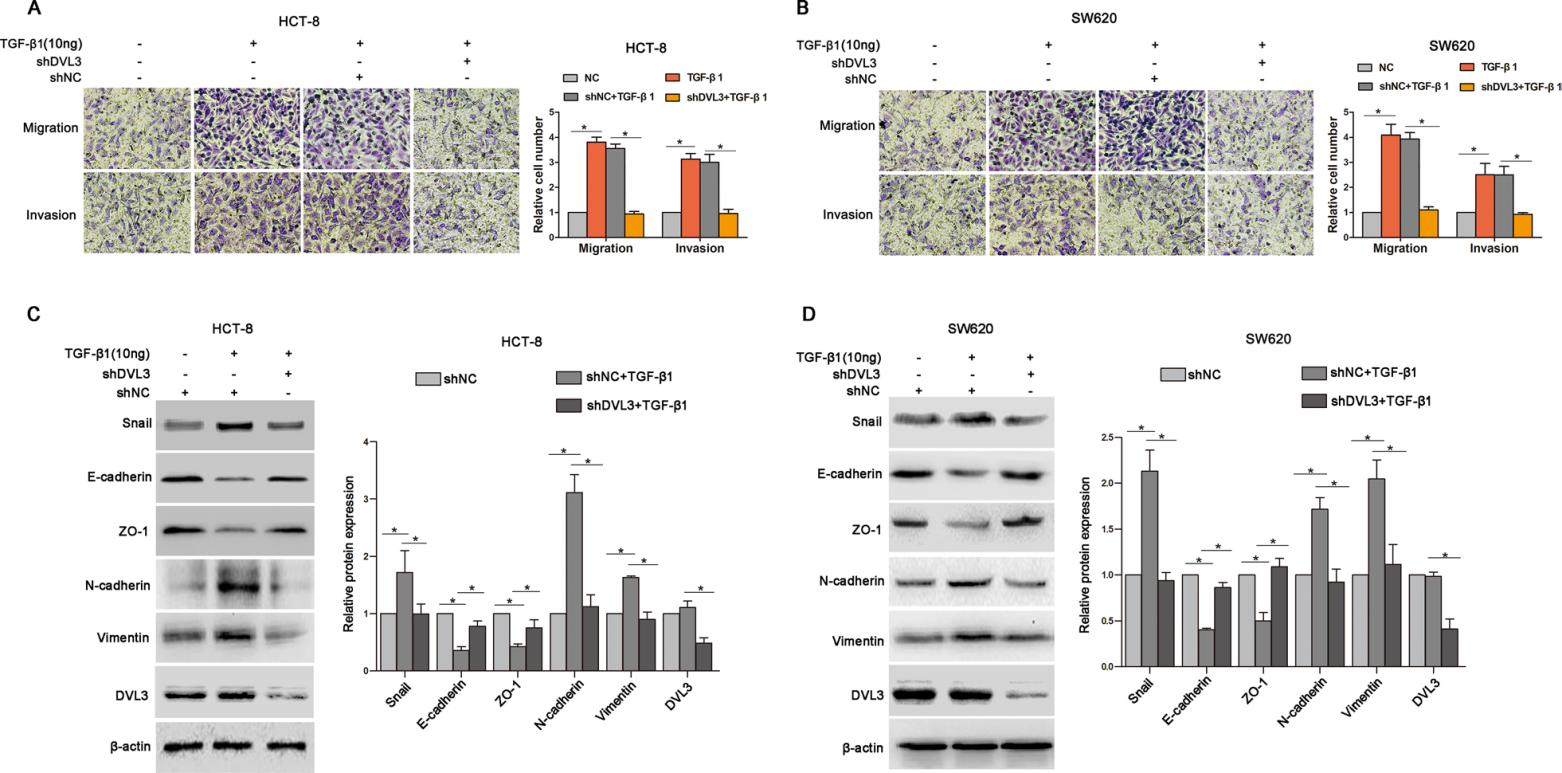
DVL3 was involved in TGF-β-induced EMT. Transwell assay showed the potential migration and invasion of HCT-8 (**A**) and SW620 (**B**) cells treated with TGF-β1 for 72 h, or transfected with shNC plus TGF-β1, or shDVL3 plus TGF-β1 for 72 h, as indicated. And then, western blotting was used to analyze the expressions of Snail, E-cadherin, ZO-1, N-cadherin, Vimentin and DVL3 in HCT-8 (**C**) and SW620 (**D**) cells transfected with shNC, shNC plus TGF-β1, or shDVL3 plus TGF-β for 72 h, as indicated. *P ≤ 0.05

To further confirm whether DVL3 plays a role in EMT induced by TGF-β1, the recombinant human TGF-β1 protein was used to treat HCT-8 and SW620 cells. The results showed that the migratory and invasive potential were elevated in CRC cells treated with TGF-β1 (10ng/mL), while TGF-β1-induced migration and invasion were inhibited by knockdown of DVL3 using shRNA (Fig. 3A, B). In addition, we found that Snail, a crucial transcriptional factor of EMT [26], was upregulated in HCT-8 and SW620 cells treated with TGF-β1, while this increase was suppressed by shDVL3. Furthermore, TGF-β1-induced downregulation of epithelial markers (E-cadherin and ZO-1), and upregulation of mesenchymal markers (N-cadherin and Vimentin) were also reversed by shDVL3 (Fig. 3C, D). These data suggested that DVL3 was involved in TGF-β1-induced EMT.

### DVL3 regulated CSLCs properties and multidrug resistance of CRC cells

**Fig. 4:**
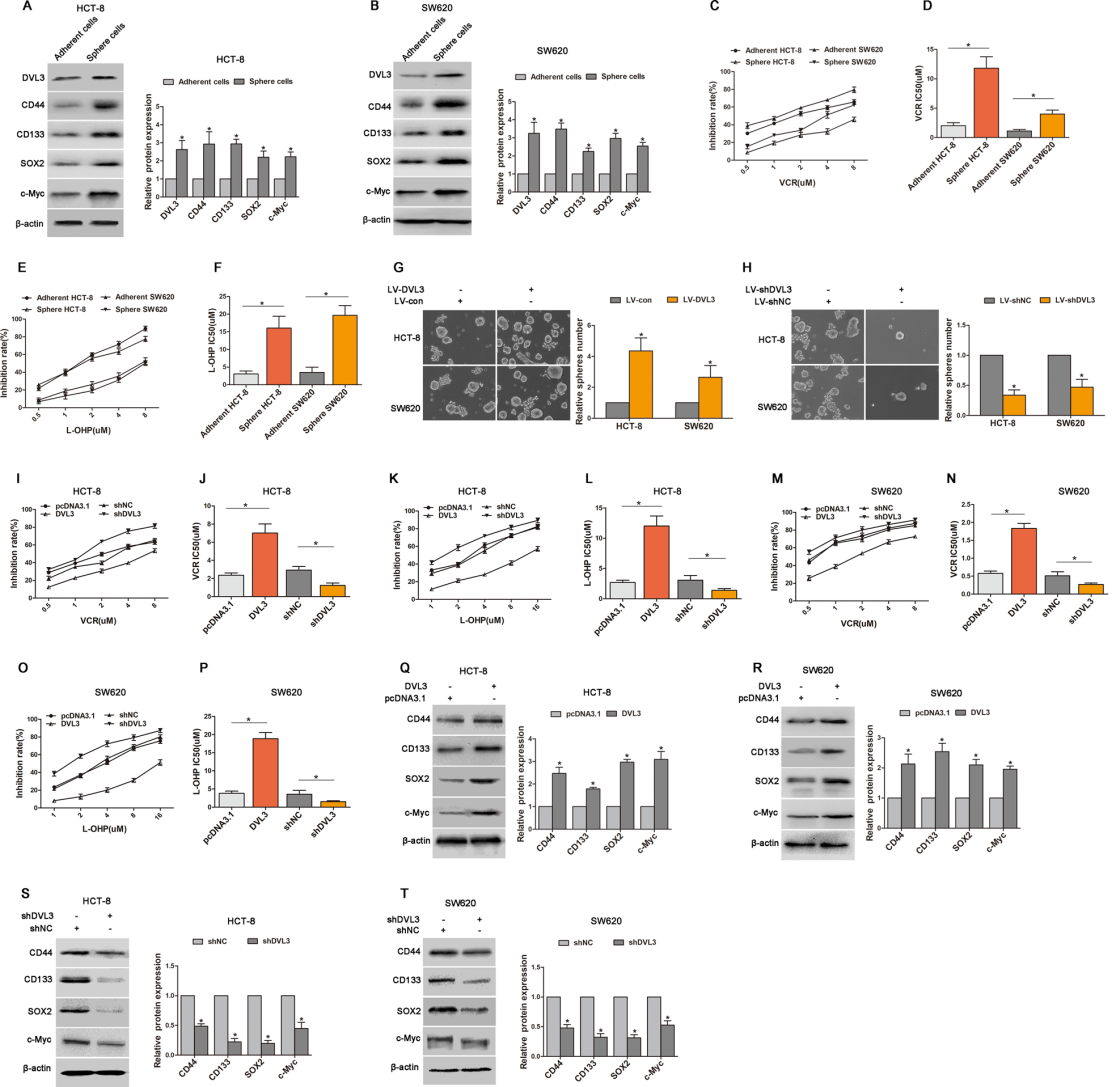
DVL3 regulated CSLCs characteristics and multidrug resistance in CRC cells. **A**, **B** The expressions of DVL3, CD44, CD133, SOX2 and c-Myc in tumorspheres and adherent CRC cells (HCT-8 and SW620) were compared by Western blotting. **C**–**F** CCK8 assay was used to check the drug sensitivities of tumorspheres and adherent CRC cells treated with VCR or L-OHP for 48 h. The IC50 values of drugs were calculated from cell survival curves using the Bliss method. **G** Tumorsphere assay was employed to determine the ability of sphere formation in CRC cells transfected with LV-con or LV-DVL3, **H** and in CRC cells transfected with LV-shNC or LV-shDVL3. **I**–**P** CRC cells were transfected with pcDNA3.1, pcDNA3.1-DVL3, shNC or shDVL3 for 24 h, followed by treatment with VCR or L-OHP for additional 48 h. And then, the drug sensitivities of CRC cells were examined by CCK8 assay, and the IC50 values of drugs were calculated from cell survival curves using the Bliss method. Western blotting presented the expressions of CD44, CD133, SOX2 and c-Myc in CRC cells transfected with pcDNA3.1 or pcDNA3.1-DVL3 (**Q**, **R**), and shNC or shDVL3 (**S**, **T**) for 72 h. *P ≤ 0.05

CSLCs has been regarded as one of the most pivotal driving force during cancer cells tumorigenesis and progression [27]. To clarify the association between DVL3 and CSLCs properties of CRC, we performed a sphere formation assay and found that the expressions of DVL3, stem cell surface markers (CD44 and CD133) and stemness-associated transcription factor (SOX2 and c-Myc) were stronger in CSLCs-enriched tumorspheres than that in monolayer-cultured HCT-8 and SW620 cells (Fig. 4A, B). In addition, the sensitivities of sphere HCT-8 and SW620 cells to vincristine (VCR IC50: 11.79 µM vs. 2.01 µM; 3.99 µM vs. 1.12 µM) and oxaliplatin (L-OHP IC50: 16.06 µM vs. 3.06 µM; 19.68 µM vs. 3.50 µM) were lower than that in adherent counterpart (Fig. 4C–F). Furthermore, the tumorspheres formation capability was promoted in HCT-8 and SW620 cells with DVL3 overexpression, while impaired in CRC cells with DVL3 knockdown (Fig. 4G, H). To further explore the function of DVL3 in chemoresistance of CRC, we checked the impact of altered DVL3 expression on sensitivities of CRC cells to VCR and L-OHP. The result revealed that the sensitivities of VCR and L-OHP were decreased in HCT-8 (IC50: 7.02 µM vs. 2.36 µM; 12.03 µM vs. 2.68 µM) and SW620 cells (1.83 µM vs. 0.58 µM; 18.87 µM vs. 3.81 µM) transfected with DVL3 recombinant vector, while improved in HCT-8 (VCR IC50: 1.24 µM vs. 2.93 µM; L-OHP IC50: 1.38 µM vs. 3.06 µM) and SW620 cells (VCR IC50: 0.27 µM vs. 0.51 µM; L-OHP IC50: 1.54 µM vs. 3.55 µM) transfected with shDVL3 (Fig. 4I–P). Consistently, overexpression of DVL3 increased the protein levels of stem cell surface markers (CD44 and CD133) and stemness-associated transcription factor (SOX2 and c-Myc) (Fig. 4Q, R), while knockdown of DVL3 played the opposite role (Fig. 4S, T). Above results suggested that DVL3 drove CSLCs phenotypic transformation and multidrug resistance of CRC.

### DVL3 promoted CSLCs and EMT phenotype of CRC via Wnt/β-catenin

**Fig. 5:**
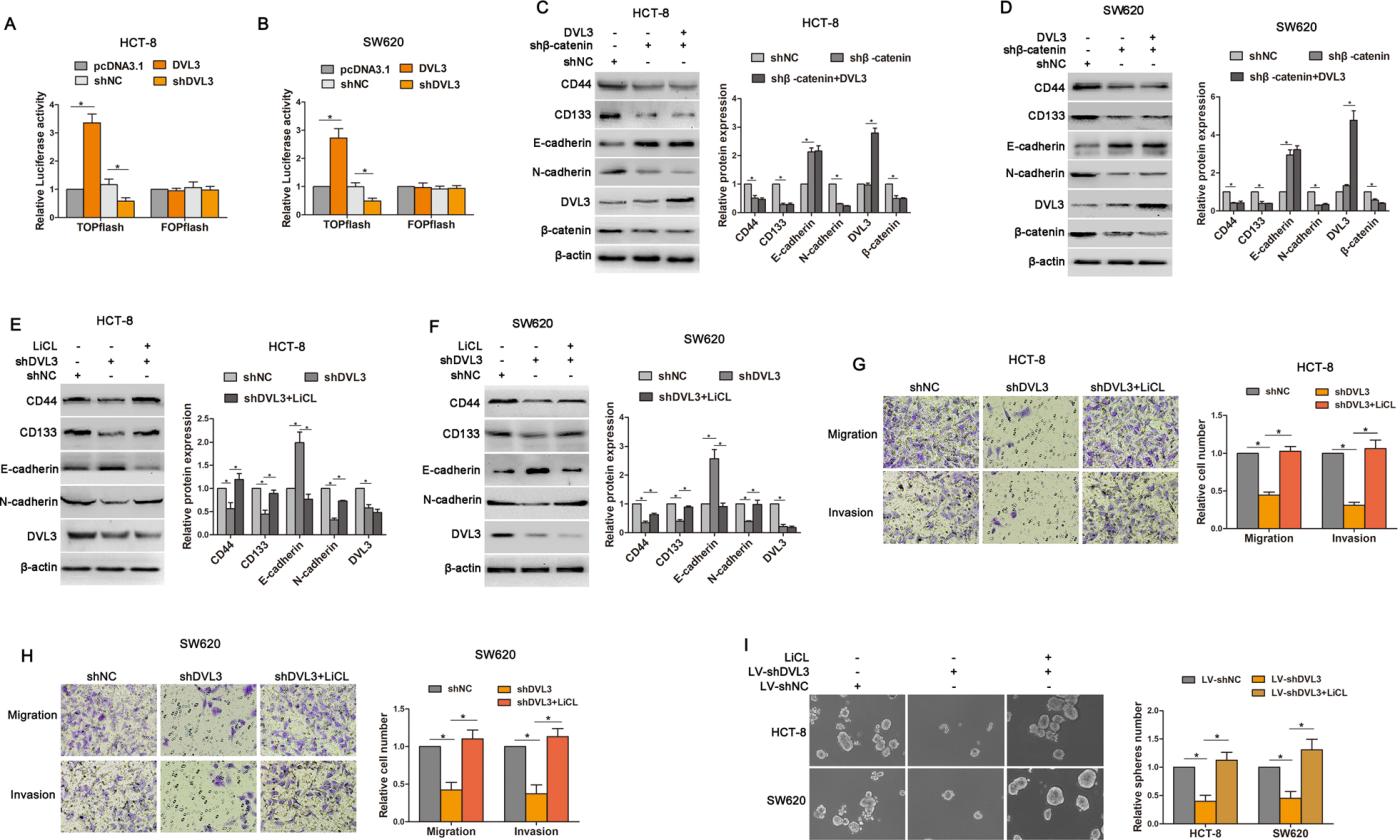
DVL3 promoted CSLCs and EMT phenotype of CRC via Wnt/β-catenin. **A**, **B** The effect of DVL3 on TOPflash and FOPflash luciferase activity were determined by a dual-luciferase reporter assay system in HCT-8 and SW620 cells. **C**, **D** The expressions of CD44, CD133, E-cadherin, N-cadherin, DVL3 and β-catenin were detected by western blotting assay in HCT-8 and SW620 cells transfected with shNC, shβ-catenin, or shβ-catenin plus pcDNA3.1-DVL3. **E**, **F** Above protein expressions were also determined by western blotting in HCT-8 and SW620 cells treated with shNC, shDVL3, or LiCL plus shDVL3 for 72 h. **G**, **H** Transwell assay was used to test the potential migration and invasion of HCT-8 and SW620 cells treated with shNC, shDVL3, or LiCL plus shDVL3 for 72 h. **I** The ability of tumorsphere formation was examined in CRC cells treated with LiCL, LV-shDVL3, or LiCL plus LV-shDVL3. *P ≤ 0.05

Growing evidence has indicated that Wnt/β-catenin plays a vital role in both stemness and mesenchymal phenotype [28], so we examined that the impact of Wnt/β-catenin signaling on DVL3-mediated CSLCs and EMT. TOPflash and FOPflash luciferase reporters, which respectively contain wildtype and mutant β-catenin/TCF-binding site, were generally used to assess the transcriptional activity of Wnt/β-catenin signaling [25]. Dual-luciferase reporter assay revealed that TOPflash luciferase activity was stronger in HCT-8 (3.36 fold) and SW620 (2.72 fold) cells transfected with DVL3 recombinant vector relative to the control group, whereas weaker in HCT-8 (50.15% reduction) and SW620 (50.84% reduction) cells transfected with shDVL3. Meanwhile, regardless of ectopic expression or knockdown of DVL3, no significant change was observed in FOPflash luciferase activity compared with the control cells (Fig. 5A, B). Moreover, after silencing β-catenin in CRC cells, the expressions of CD44 and CD133 were decreased. EMT-like molecular changes were also inhibited in HCT-8 and SW620 cells transfected with shβ-catenin, this was confirmed by E-cadherin upregulation and N-cadherin downregulation. However, DVL3 lost the ability to regulate above protein expressions when β-catenin was silenced (Fig. 5C, D). These results indicated that Wnt/β-catenin signaling was involved in DVL3-mediated CSLCs and EMT.

Based on above observations, we further assessed whether activation of Wnt/β-catenin was able to reverse the repressive role of DVL3 knockdown in EMT and CSLCs. The result showed that shDVL3-induced downregulations of CD44, CD133 and N-cadherin, and upregulation of E-cadherin were eliminated by treatment of LiCl, which is known as activator of Wnt/β-catenin pathway (Fig. 5E, F). Furthermore, we found that shDVL3-inhibited migration, invasion, and tumorspheres formation were reversed in HCT-8 and SW620 cells treated with LiCl (Fig. 5G–I). Above data suggested that DVL3 drove CSLCs and EMT phenotypes in a Wnt/β-catenin signaling dependent manner.

### Wnt/β-catenin/c-Myc/SOX2 was critical for DVL3-promoted CSLCs characteristics and EMT in CRC

**Fig. 6:**
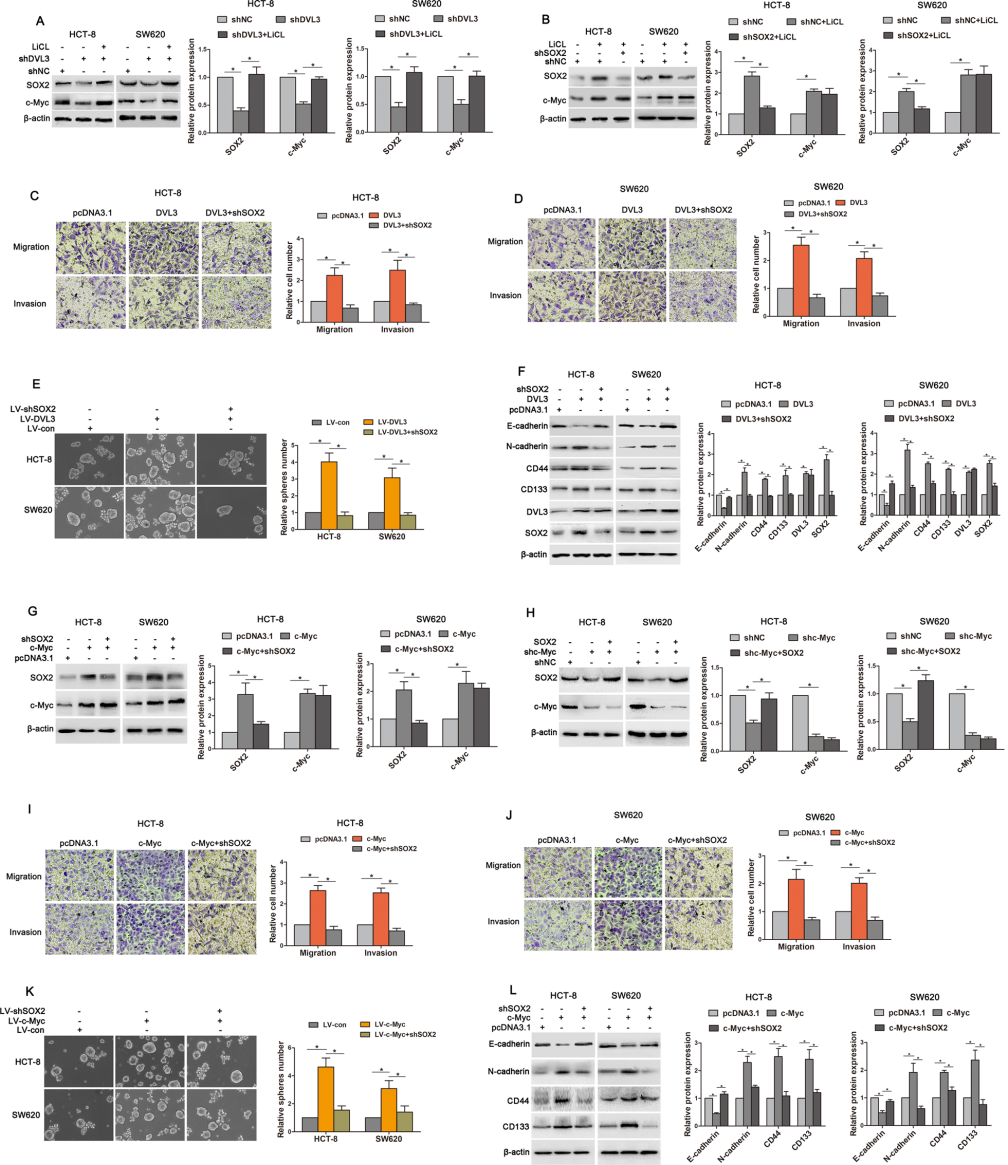
Wnt/β-catenin/c-Myc/SOX2 was critical for DVL3-promoted CSLCs characteristics and EMT of CRC. **A** HCT-8 and SW620 cells were treated with shNC, shDVL3, or LiCL plus shDVL3 for 72 h, **B** while treated with shNC, LiCL plus shNC or shSOX2 for 72 h. Subsequently, the expressions of SOX2 and c-Myc were determined by western blotting. **C**, **D** HCT-8 and SW620 cells were transfected with pcDNA3.1, pcDNA3.1-DVL3, or pcDNA3.1-DVL3 plus shSOX2 for 72 h. And then, the potential migration and invasion of these cells were examined by transwell assay. **E** The ability of sphere formation was examined by tumorsphere assay in CRC cells treated with LV-con, LV-DVL3, or LV-shSOX2 plus LV-DVL3. **F** Western blotting showed the expressions of E-cadherin, N-cadherin, CD44, CD133, DVL3 and SOX2 in CRC cells transfected with pcDNA3.1, pcDNA3.1-DVL3, or shSOX2 plus pcDNA3.1-DVL3 for 72 h. **G** Western blot analysis of SOX2 and c-Myc were performed in CRC cells transfected with pcDNA3.1, pcDNA3.1-c-Myc, or shSOX2 plus pcDNA3.1-c-Myc for 72 h, **H** as well as in CRC cells transfected with shNC, shc-Myc, or pcDNA3.1-SOX2 plus shc-Myc for 72 h. **I**, **J** Transwell assay was used to evaluate the potential migration and invasion of CRC cells transfected with pcDNA3.1, pcDNA3.1-c-Myc, or shSOX2 plus pcDNA3.1-c-Myc for 72 h. **K** Tumorsphere assay was performed in CRC cells treated with LV-con, LV-c-Myc, or LV-shSOX2 plus LV-c-Myc. **L** The protein levels of E-cadherin, N-cadherin, CD44 and CD133 were detected by western blotting in CRC cells transfected with pcDNA3.1, pcDNA3.1-c-Myc, or shSOX2 plus pcDNA3.1-c-Myc for 72 h. *P ≤ 0.05

It has been reported that SOX2 is involved in stemness, chemoresistance, metastasis and recurrence of cancer [29]. Therefore, we asked whether SOX2 is required for DVL3-promoted CSLCs characteristics and EMT in CRC cells. We found that although expressions of SOX2 and c-Myc (a target gene of Wnt/β-catenin) were reduced in HCT-8 and SW620 cells transfected shDVL3, this reduction was rescued by LiCl (Fig. 6A). It is true that LiCl can upregulated SOX2 and c-Myc. However, LiCl-increased expression of SOX2 was reversed by shSOX2 despite LiCl-increased expressions of c-Myc was not affected (Fig. 6B). This data indicated that DVL3 increased SOX2 expression via Wnt/β-catenin. Moreover, DVL3-mediated migration, invasion and sphere formation of CRC cells were abolished by shSOX2 (Fig. 6C–E), and DVL3-induced downregulation of E-cadherin, and upregulations of N-cadherin, CD44 and CD33 were also reversed by knockdown of SOX2 (Fig. 6F). These results suggested that DVL3 enhanced Wnt/β-catenin/SOX2 to promote CSLCs characteristics and EMT in CRC.

We next identified the underlying mechanism that DVL3 upregulated SOX2 via Wnt/β-catenin. In Wnt/β-catenin signaling pathway, T cell factor/Lymphoid enhancer factor (TCF/LEF) transcription factors activate target gene via binding to special promoter region [30]. Although SOX2 expression was regulated by Wnt/β-catenin signaling in this study, there is no binding site matched with TCF/LEF in the promoter region of SOX2 [31], which hints that Wnt/β-catenin signaling do not control SOX2 directly and the downstream target gene may mediate SOX2 expression. It has been reported that c-Myc, a directly binding target gene of Wnt/β-catenin downstream, can activate transcription of SOX2 via binding to SOX2 promoter [32]. So, we further evaluated the role of c-Myc in regulating SOX2 expression of CRC cells. The result showed that SOX2 expression was increased or reduced in HCT-8 and SW620 cells with c-Myc overexpression or knockdown, respectively. However, c-Myc expression was not significantly changed by SOX2 deficiency or ectopic expression (Fig. 6G, H), and LiCL-induced c-Myc expression was also not affected by SOX2 (Fig. 6B). This suggested that c-Myc may exert regulation of SOX2 expression in CRC cells. Furthermore, we found that the potentials of migration, invasion and sphere formation were increased in CRC cells transfected with c-Myc recombinant vector, while this increasements of migration, invasion and tumorsphere formation were reversed by shSOX2 (Fig. 6I–K). As expect, c-Myc-inhibited expression of E-cadherin, and c-Myc-increased expressions of N-cadherin, CD44 and CD133 were also abolished by silencing SOX2 (Fig. 6L). Above data suggested that DVL3 enhanced Wnt/β-catenin/c-Myc to control the expression of SOX2, augmenting CSLCs characteristics and EMT in CRC cells.

### Silencing DVL3 impaired tumorigenicity of CRC cells in vivo

**Fig. 7:**
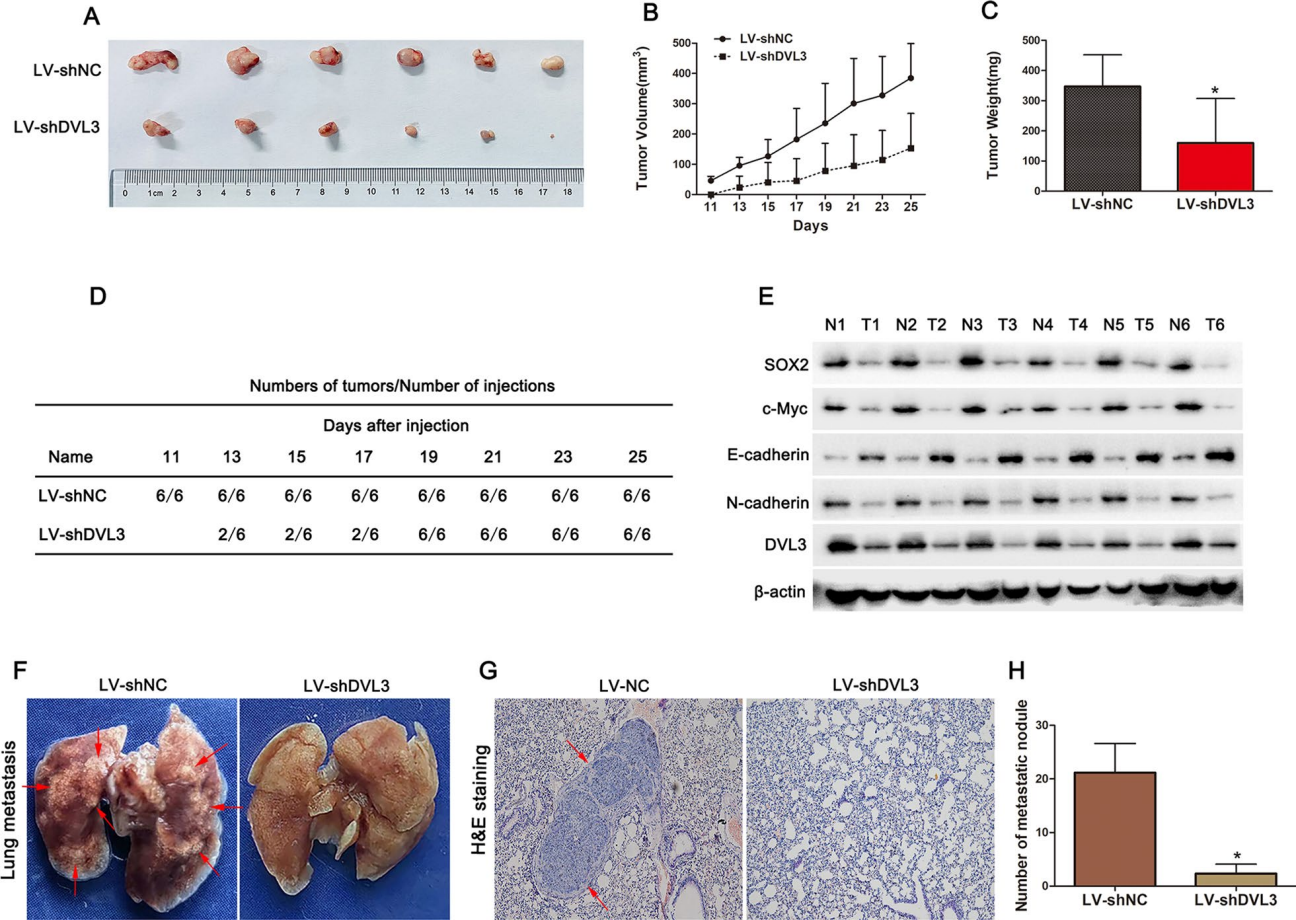
Silencing DVL3 inhibited tumorigenicity and metastasis of CRC cells in vivo. **A** For tumor xenograft assay in vivo, HCT-8 cells stably expressing LV-shDVL3 or LV-shNC were injected subcutaneously into nude mice. The representative images of tumor xenografts are shown. **B** Tumor growth curves of tumors in nude mice injected with HCT-8 cells stably expressing LV-shDVL3 or LV-shNC. Once the tumors became palpable, tumor volumes were measured with Vernier calipers every two days. **C** After the nude mice were killed, tumors xenograft were excised and weighed. **D** Tumors of LV-shDVL3 group formed at an earlier time point and developed at a faster rate than those of LV-shNC group. **E** The expression of SOX2, c-Myc, E-cadherin, N-cadherin and DVL3 were determined by western blotting in tumor xenograft with LV-shDVL3 or LV-shNC. **F** Representative images of lung metastasis and **G** H&E staining of metastatic tumors in nude mice injected with HCT-8 cells stably expressing LV-shDVL3 or LV-shNC via tail vein. **H** The statistical data of lung metastasis that developed in the mouse model. *P ≤ 0.05

To verify the in vivo role of DVL3, we constructed CRC cells with stable knockdown of DVL3. And then, HCT-8 cells with or without stable knockdown of DVL3 were subcutaneously injected into BALB/c nude mice to assess tumorigenicity. Tumor development was measured once every two days in xenograft mouse model. As shown in Fig. 7A and B, tumor volume was inhibited by silencing DVL3 in vivo compared to the control. After 25 days of injection, the mice were sacrificed, and the tumors were weighed. The data demonstrated that the tumors formed with DVL3-silencing CRC cells were lighter than those formed with the control cells (Fig. 7C). Moreover, the data presented in Fig. 7D showed that the first palpable tumor and tumor development in 100% (6/6) in DVL3-silencing group appeared 2 and 8 days earlier than those in the control, respectively. Furthermore, the expressions of SOX2, c-Myc and N-cadherin in DVL3-silencing tumors were lower than those in the control tumors, while the opposite trend was observed for E-cadherin expression (Fig. 7E). Interestingly, the fewer and smaller lung metastatic nodules were also found in nude mice injected with DVL3-silencing cells through tail vein for 4 weeks, compared to the mice injected with LV-shNC (Fig. 7F–H). Above results suggested that silencing DVL3 delayed tumorigenesis and metastasis of CRC cells in vivo.

## Discussion

CRC is a widespread malignant solid tumor, which represents approximately 10% of all cancers [33], and remains the second primary reason of cancer-related mortality worldwide. Therefore, CRC is a global public health challenge including morbidity, mortality, health care services utilization and growingly high medical costs [34]. Although CRC treatment has growingly advanced over the recent decades, the therapy effect is unsatisfactory and the 5-year survival has slowly increased [35]. Several studies have reported that metastasis and drug-resistance remain major obstacles limiting the effective treatment of CRC [36, 37]. Recently, EMT and CSLCs have become widely identified as pivotal biological processes contributing to cancer cell metastatic spread and therapeutic resistance from primary tumor [10, 38]. Therefore, it is important to understanding the molecular mechanism underlying regulation of EMT and CSLCs for improving therapeutic strategies and prognosis of CRC patient.

It has been reported that DVL, as a key component of Wnt signaling, performs a crucial function in stem cell development and adult tissue homeostasis [39], and its deregulation results in human development disorders and other diseases including malignant tumor [40, 41]. Our study identified that DVL3, one of DVL family members, was overexpressed in primary tumor tissue and multiple cell lines of CRC. Moreover, the ectopic expression of DVL3 was also found in CRC tissue with lymph nodal metastasis, and negatively correlated with the survival including OS, DFS and DSS. These data suggested that DVL3 may serve as a potential biomarker to predict poor prognosis of CRC patients. However, the role and mechanism of DVL3 in CRC progression including EMT and CSLCs remain largely unknown.

EMT is a biological process in which epithelial cells lose cell-cell adhesion and cell polarity and acquire mesenchymal features [42]. EMT has emerged as a key regulator of metastasis and recurrence by facilitating tumor cell motility and invasion in many cancers [43, 44]. CSLCs possess normal stem cell traits including self-renewal and multipotency, which lead to tumorigenicity, relapse, treatment resistance and metastasis [45]. Although the number of CSLCs in tumor tissue is very small, CSLCs can withstand killing effects of chemotherapy through a series of defensive lines [46, 47]. The tumor volume may be reduced by conventional chemotherapeutics, nevertheless the residual CSLCs can seed new tumor tissue by virtue of their powerful self-renewal ability [48]. In addition, it has been found that the drug-resistant cancer cells frequently are endowed with characteristics of CSLCs during process of chemotherapy [49]. In essence, CSLCs have been regarded as the “roots” of aggressive tumors.

Notably, the activation of EMT process has been involved in stem cell traits for both normal and cancer cells [50]. Cells with an EMT phenotype effect molecular characteristics of CSLCs, and EMT also plays an essential role in CSLCs enrichment and therapeutic resistance [51, 52]. Therefore, targeting key molecule in EMT and CSLCs has become a frontier hotspot of cancer therapy. In this study, we found that DVL3 positively regulated migration, invasion and EMT-like molecular changes, while silencing DVL3 abolished TGF-β1-induced EMT. Moreover, DVL3 was also found to be ectopically expressed in tumorshere of CRC cells compared with adherent cells. DVL3 overexpression promoted tumorshere formation, and drug-resistance of CRC cells to VCR and L-OHP, while knockdown of DVL3 inhibited the tumorshere formation and improved the drugs sensitivities. Furthermore, DVL3 positively controlled the expressions of stem cell surface markers including CD44 and CD133, and upregulated the expressions of stemness-associated transcription factors such as SOX2 and c-Myc. These data suggested that DVL3 drove EMT and CSLCs phenotypes, and enhanced metastasis and multi-resistance of CRC. However, the underlying mechanism by which DVL3 regulated EMT and CSLCs remains unclear in CRC cells.

To elucidate how DVL3 promoted EMT and CSLCs, we determined effect of DVL3 on Wnt/β-catenin signaling, which is simultaneously responsible for maintenance of EMT and stemness [53]. It is reported that almost all sporadic CRCs are linked to abnormal Wnt/β-catenin signaling, whose excessive activation can increase complex formation of β-catenin and TCF/LEF to trigger downstream target gene expression [54]. This pathway dysregulation has been engaged in a series of biological processes in cancers, including proliferation, apoptotic escape, and cell metastasis and drug resistance [55]. Here, we employed TOPflash and FOPflash luciferase reporters, which respectively contain the wildtype and mutant β-catenin/TCF-binding site for characterizing transcriptional activation of β-catenin/TCF [25], to check the impact of DVL3 on activity of Wnt/β-catenin signaling. The data acquired from dual-luciferase reporter assay showed that the overexpression and knockdown of DVL3 respectively amplificated and impaired TOPflash activity, while DVL3 did not significantly changed FOPflash activity. This suggested that DVL3 can enhanced activity of Wnt/β-catenin in CRC cells. Consistent with our research, previous study has also revealed that even in absence of Wnt ligand, DVL can potently activate Wnt/β-catenin signaling [56]. It is well known that β-catenin is one of pivotal transducer in canonical Wnt signaling [57]. Here, we found that silencing β-catenin deprived the ability of DVL3 to induce EMT-like molecular changes and stem cell marker expression, indicating that DVL3 triggered Wnt/β-catenin signaling to drive EMT and CSLCs of CRC. So, We speculated that Wnt/β-catenin activation could abolish inhibition of EMT and CSLCs mediated by silencing DVL3. LiCl is widely used as the potent activator of the Wnt/β-catenin signaling [58]. Our result showed that LiCl restored shDVL3-reduced EMT-like molecular changes and stem cell marker expression, and reversed shDVL3-inhibited migration, invasion and tumorsphere formation in CRC cells. These data further confirmed that DVL3 promoted EMT and CSLCs properties via Wnt/β-catenin signaling, while the underlying mechanism needs to be further clarified.

SOX2 is an essential transcription factor for embryonic development and plays a vital function in sustaining stemness of embryonic cells and various adult stem cell populations. Its importance is confirmed by the fact that SOX2-deficient embryos die straight after implantation [59]. Moreover, dysregulation of SOX2 also functions as a critical factor contributing to cancer pathogenesis. Accumulating evidence has shown amplification of SOX2 gene locus and overexpression of SOX2 that in turn motivates cancer progression, including tumor initiation, EMT, metastasis, recurrence, CSLCs phenotype transformation and drug resistance [60]. In gliomas, SOX2 expression strengthens migration and invasion, and is positively correlated with malignancy grade [61]. Beyond that, there is a tendency for a lower survival rate for SOX2-overexpressing patients with ovarian cancer and non-small cell lung cancer [62]. In addition, studies have emphasized the central roles of Wnt/β-catenin in maintenance of EMT and stemness, which is achieved by regulating SOX2 in cancer cells [63]. Considering that DVL3 increased the activity of Wnt/β-catenin and expression of SOX2 in this study, we hypothesized that DVL3 enhanced EMT and CSLCs traits via Wnt/β-catenin/SOX2 axis. Consistent with this hypothesis, we found that DVL3 increased the expression of SOX2 via Wnt/β-catenin signaling, and silencing SOX2 reversed DVL3-induced metastasis, tumorsphere formation, EMT-like molecular changes and stemness marker expressions.

Although our results revealed that Wnt/β-catenin was responsible for DVL3-induced expression of SOX2 in CRC cells, it has been reported that SOX2 is not direct target gene of Wnt/β-catenin [31]. Numerous studies have shown that the binding of β-catenin/TCF/LEF transcription complex to Wnt responsive DNA elements is required for activating target gene expression [64]. However, there is no evidence showing that TCF/LEF can bind to the promoter region of SOX2, which suggests that Wnt/β-catenin may indirectly mediate expression of SOX2. It is well known that c-Myc is one of direct target genes in Wnt/β-catenin signaling downstream, and involved in both EMT and CSLCs traits in the progression of cancers including breast and liver cancer [65, 66]. Interestingly, recent studies have confirmed that c-Myc functions as a transcriptional activator of SOX2 by binding to SOX2 gene promoter, and cooperates with SOX2 to maintain stemness and tumorigenicity in breast and lung cancer [32]. Here, we found that DVL3 upregulated c-Myc and SOX2 via Wnt/β-catenin, and shSOX2 eliminated LiCL-induced expression of SOX2, without altering LiCL-induced expression of c-Myc in CRC cells. In addition, c-Myc overexpression and knockdown increased and reduced expression of SOX2 respectively, but this changed expression of c-Myc was not regulated by SOX2. Above results suggested that DVL3 upregulated SOX2 by Wnt/β-catenin/c-Myc axis. Furthermore, c-Myc facilitated metastasis, tumorsphere formation, EMT-like molecular changes and stemness marker expressions in CRC cells, while shSOX2 blocked these facilitation exerted by c-Myc. As expected, in BALB/c nude mice, silencing DVL3 also inhibited tumorigenicity, metastatic potential and expressions of c-Myc, SOX2 and N-cadherin in CRC cells, as well as upregulated E-cadherin. Collectively, above data suggested that DVL3 enhanced EMT and CSLCs characteristic via Wnt/β-catenin/c-Myc/SOX2 axis, which provided a new strategy for successful CRC treatment.

There are still some limitations in our study. First, due to the multiple functional domains of DVL3, it can bridge the transduction of multiple signaling including TGF-β, Notch and Hedgehog, besides Wnt signaling [19, 67, 68]. However, it remains unclear whether DVL3 integrates these signaling to synergistically regulate EMT and CSLCs of CRC. Moreover, this study suggested that targeting DVL3 may be a promising therapeutic option for CRC patient. Drugs targeting DVL3 may improve the treatment effect of CRC. Although universal inhibitors of the DVL family have been developed, due to the lack of drugs specifically targeting DVL3, we could not examine the cooperative effect of DVL3 targeting inhibitors and CRC chemotherapeutics.

## Conclusion

Taken together, this study has presented evidences to reveal the role and underlying mechanism of DVL3 in EMT and CSLCs of CRC. DVL3 was overexpressed in CRC tissues and tumorsphere with stem cell properties, and positively related to poor prognosis of CRC patients. Mechanistically, DVL3 promoted metastasis and multidrug resistance, and maintained EMT and CSLCs phenotype via Wnt/β-catenin/c-Myc/SOX2 axis. Furthermore, silencing DVL3 decreased tumorigenicity and metastasis of CRC cells in vivo. Overall, the acquired data indicated that DVL3 may be a potential marker for poor prognosis and a target for drug therapy in CRC. In future studies, more exploration is required to identify the regulatory network bridged by DVL3 in EMT and CSLCs. Moreover, it is necessary to screen specific drugs targeting DVL3 and clarify its role and mechanism in improving therapeutic effect of CRC.

## Data Availability

The data that support the findings of this study are available from the corresponding author upon reasonable request.

## Abbreviations

CRC: Colorectal cancer
EMT: Epithelial-to-mesenchymal transition
CSLCs: Cancer stem-like cells
Disheveled3: DVL3
OS: Overall survival
DFS: Disease free survival
DSS: Disease-specific survival
SOX2: Sex determining region Y box transcription factor 2
c-Myc: Cellular proto-oncogene v-Myc myelocytomatosis viral oncogene homolog
ZO-1: Zona Occludens 1
Wnt: Wingless-type MMTV integration site family
TGF-β1: Transforming growth factor beta 1
VCR: Vincristine
L-OHP: Oxaliplatin
TCF/LEF: T cell factor/Lymphoid enhancer factor
LiCL: Lithium chloride
